# Biodegradation of Polystyrene by Plastic-Eating Tenebrionidae Larvae

**DOI:** 10.3390/polym16101404

**Published:** 2024-05-15

**Authors:** Erika Alessia Di Liberto, Giuseppe Battaglia, Rosalia Pellerito, Giusy Curcuruto, Nadka Tz. Dintcheva

**Affiliations:** 1 Dipartimento di Ingegneria, Università di Palermo, Viale delle Scienze, Ed. 6, 90128 Palermo, Italy; giuseppe.battaglia03@unipa.it; 2 Istituto Comprensivo Statale “Luigi Capuana”, Via A. Narbone, 55, 90138 Palermo, Italy; rosannapellerito@libero.it; 3 Institute for Polymers, Composites and Biomaterials (IPCB)—CNR, Via Paolo Gaifami 18, 95126 Catania, Italy; giusy.curcuruto@cnr.it

**Keywords:** biodegradation, polystyrene, *Tenebrio Molitor*, *Zophobas Morio*

## Abstract

Polystyrene (PS) is an extremely stable polymer with a relatively high molecular weight and a strong hydrophobic character that makes it highly resistant to biodegradation. In this study, PS was subjected to biodegradation tests by *Tenebrio Molitor* (*T. Molitor*) and *Zophobas Morio* (*Z. Morio*) larvae. Specifically, six different experimental diets were compared: (i) *T. Molitor* fed with bran; (ii) *T. Molitor* fed only PS; (iii) *T. Molitor* fed only PS treated with H_2_O_2_; (iv) *Z. Morio* fed with bran; (v) *Z. Morio* fed only PS; and (vi) *Z. Morio* fed only PS treated with H_2_O_2_. Therefore, the mass change of the larvae and the survival rate were measured periodically, while the frass collected after 15 and 30 days was analyzed by different analyses, such as spectroscopy (FTIR), spectrometry (molecular weight and polydispersity), thermal analysis (TGA) and microscopy (scanning electron microscopy observations). The obtained results suggest that in the case of *T. Molitor* larvae, larvae feeding on bran showed the highest survival rate of ~94% at 30 days, while in the case of the *Z. Morio* larvae, the highest survival rate was exhibited by larvae eating PS-H_2_O_2_. Although not strongly pronounced, the M_w_ and M_n_ of PS in the frass of both *T. Molitor* and *Z. Morio* larvae decreased over 30 days, suggesting PS biodegradation. Finally, the morphological analysis shows that PS samples isolated from the frass of *T. Molitor* and *Z. Morio* larvae showed completely different, rough and irregularly carved surface structures, in comparison to PS before biodegradation.

## 1. Introduction

Plastic products are widely used around the world because of their low cost and ease of production. Presently, over 400 million tons (Mt) of plastics are produced yearly, with exponential growth over the past 50 years, and more than 360 million tons of polymers produced per year are of fossil-based [1]. Specifically, the main products are polyethylene (PE), polypropylene (PP), polyethylene terephthalate (PET), polystyrene (PS) and polyvinyl chloride (PVC), materials that have very attractive characteristics, such as low density, good mechanical impact stability and resistance to chemicals and corrosion, and are commonly used for packaging, construction, automotives, agriculture and electronic devices. The increasing world population has led to increased production and consumption of plastic materials. However, the accumulation of petrol-plastic wastes in the environment has become the focus of worldwide attention because of the environmental problems caused by their improper disposal after use and decommissioning and because the natural degradation of plastics is very sluggish, resulting in the accumulation of plastic waste that poses a serious environmental threat [2,3,4]. 

The main disposal methods include landfill, incineration, chemical treatment and recycling [5,6]. Landfilling is the process of disposing and accumulating waste in a certain area. It is a traditional method of waste management, which is widely used in many parts of the world. However, major environmental issues like global warming and the increase in soil acid grow as CO_2_ and other gasses are emitted from landfills. This method destroys soil, affects groundwater and cannot effectively degrade waste plastics. Incineration is the process of burning waste in an incinerator until it is converted to ashes and gas. The process produces a large number of toxic gases, which volatilize into the air and cause harmful effects to public health, biodiversity and ecosystems. Furthermore, the cost of chemical treatment and recycling is high. Hence, these methods are not enough to successfully solve the problem of environmental pollution from plastic products [6,7].

Considering that a lot of plastic waste has not been scientifically or properly treated, through a series of physical and chemical processes, microplastics are formed and dispersed into the environment [3,8]. Furthermore, organisms can interact with plastic waste: several species of vertebrates and invertebrates have been reported to ingest or become entangled in plastic as animals are unable to distinguish food from plastic in the environment, resulting in the ingestion of plastic particles [9,10]. 

In recent years, several studies have explored the unusual ability of some insects to consume and even biodegrade different types of plastics. While feeding, insects come into contact with a wide range of hydrocarbon polymers in their diet, and the gut of some insects contains microbial symbionts that aid in decomposing these polymers. Recently, various insects, such as mealworms, meal beetles, weevils or wax moths, particularly of the orders Coleoptera and Lepidoptera, were identified as having remarkable abilities to consume and degrade a wide range of synthetic polymers such as polyethylene, polyurethane, polypropylene, polystyrene and polyvinyl chloride into lower-molecular-weight, simple and nontoxic molecules, which are eventually excreted as fecula [11]. Furthermore, the gut microbiota of these organisms, which is involved in the plastic degradation process, has been investigated in recent studies [12,13,14,15]. 

Moreover, the rate of plastic consumption by these insects is higher than that of bacteria and fungi, isolated from various sources, such as soil, garbage or sewage sludge. These insects mainly include the yellow mealworm (larvae of *Tenebrio Molitor*), the greater wax moth (larvae of *Galleria Mellonella*) and the superworm (larvae of *Zophobas Morio*), among others [5]. The complete life cycle of Lepidopterans and Coleopterans insects consists of four stages: eggs, larvae, pupae and adults; a significant portion of their life is spent in the larval stage. It is worth noting that the length of each stage of the insect life cycle can vary according to several factors, such as temperature, humidity, nutrition and age of the parents. Interestingly, the initial larval and pupal stages of *Tenebrio Molitor,* also named yellow mealworm larvae for their color, are rich in protein and considered a popular dish in some countries. The larval period varies from 22 to 100 days, while the pupal period lasts about 8 days. *Zophobas Morio*, a synonym of *Zophobas Atratus*, is commonly known as a superworm or royal worm, associated with damaged stored foods. *Z. Atratus* adult beetles look very similar to *T. Molitor*, but are larger and measure about 2–3 cm in length [12,16]. These larvae can be easily raised on fresh oats, wheat bran or cereals with potatoes, cabbage, carrots or apple.

Polystyrene (PS), molecular formula [−CH(C_6_H_5_)CH_2_−]_n_, commonly known as Styrofoam, accounted for about 5.3% (c.a. 21 Mt/year) of the total plastic consumption in 2022 [17]. Although PS is considered a durable plastic, PS products are often designed for a very short service time and one-time use because of the low cost of this material [18]. It is an extremely stable polymer with a high molecular weight and a strong hydrophobic character, which makes this polymer highly resistant to biodegradation. Several soil invertebrates have also been tested to determine whether they were able to degrade PS, including earthworms, isopods, slugs, millipedes and snails [19].

In this work, the larvae of the yellow mealworm *Tenebrio Molitor* and superworm *Zophobas Morio*, two species of Coleopterans Tenebrionidae larvae, were chosen and prepared to carry out the research. Before the tests, the larvae were placed in a polypropylene plastic container and fed their usual food, then expanded PS foam was used as a raw material for the larvae of both species. 

The degradation of polystyrene treated with hydrogen peroxide and subjected to microwave irradiation was also studied. The use of microwaves for polymer degradation is an excellent alternative to conventional thermal heating, offering increased reaction rates, reduced reaction times and energy savings. Typically, polymers such as PS have poor dielectric properties and are unable to absorb enough microwave energy to achieve the necessary temperature for degradation. Consequently, in order to increase absorption, it is necessary to use solvents such as hydrogen peroxide that can absorb microwave energy to achieve the required temperature [20,21,22]. The combination of hydrogen peroxide and microwaves may make polystyrene more attractive to eat. In this study, we focused on the chemical–physical characterization of the material and not on the identification of microrganisms that are able to degrade polystyrene. In particular, two species of insect larvae were fed polystyrene (PS) and polystyrene treated with hydrogen peroxide (PS-H_2_O_2_) as the sole diet to determine and compare their feeding ability and survival rates; in addition, changes in the product properties of the larvae after feeding PS and PS-H_2_O_2_ were analyzed. Six different experimental diets were compared: (i) *T. Molitor* fed with bran; (ii) *T. Molitor* fed only PS; (iii) *T. Molitor* fed only PS treated with H_2_O_2_; (iv) *Z. Morio* fed with bran; (v) *Z. Morio* fed only PS; and (vi) *Z. Morio* fed only PS treated with H_2_O_2_. The change in the larvae mass and the survival rate were measured periodically. Furthermore, a morphological analysis of frass was performed by scanning electron microscopy (SEM); M_n_, M_w_ and polydispersity index (PDI) were determined by Size-Exclusion Chromatography (SEC); and additional characterization of the residual polymer was obtained using Thermogravimetric Analysis (TGA) and Fourier Transform Infrared Spectroscopy (FTIR) to identify chemical modifications resulting from PS digestion. An innovative aspect has been investigated regarding the treatment of PS with H_2_O_2_ in order to facilitate PS biodegradation by Tenebrionidae larvae.

## 2. Materials and Methods

### 2.1. Test Materials

*Tenebrio Molitor* (yellow mealworms) and *Zophobas Morio* (superworms) at the larval stage were purchased from a local supplier (Zoo Service, Palermo, Italy) and ranged in length from 2 to 3 cm and from 4 to 8 cm, respectively. Expanded PS foam waste from electronic equipment boxes was collected and used as a feedstock for the larvae of both species. The number-averaged molecular weight (M_n_) and weight-averaged molecular weight (M_w_) of PS were 133,174 Da and 236,693 Da, respectively. Hydrogen peroxide (36 volumes 100 mL) was purchased from a local supplier (Sferlazzo Pharmacy, Palermo, Italy). For microwave oxidation, a block of PS was placed in a beaker with hydrogen peroxide H_2_O_2_ for 5 min and then microwaved into a commercial microwave oven at 800 Watt for 3 min, which caused the initial breakage of the polystyrene chains. 

### 2.2. Feeding Tests

To compare the PS consumption and biodegradation between the larvae of the two species, six treatments were prepared based on feeding conditions. A group of *T. Molitor* and *Z. Morio* larvae (30 as a group) were reared on PS foam (~0.4 g) and PS foam treated with H_2_O_2_ (PS H_2_O_2_) (~0.4 g) as their exclusive diet in a polypropylene plastic container. As a control, other groups of larvae (30 as a group) were reared on a normal diet of wheat bran and fruits. Prior to the commencement of the feeding experiment, the larvae were subjected to a 48 h starvation period to empty their intestines. All containers were maintained in the climatic chamber under controlled conditions, i.e., 25 ± 1 °C, 60 ± 2% humidity, for a period of 30 days.

### 2.3. Collection and Characterization of Frass

**Figure 1:**
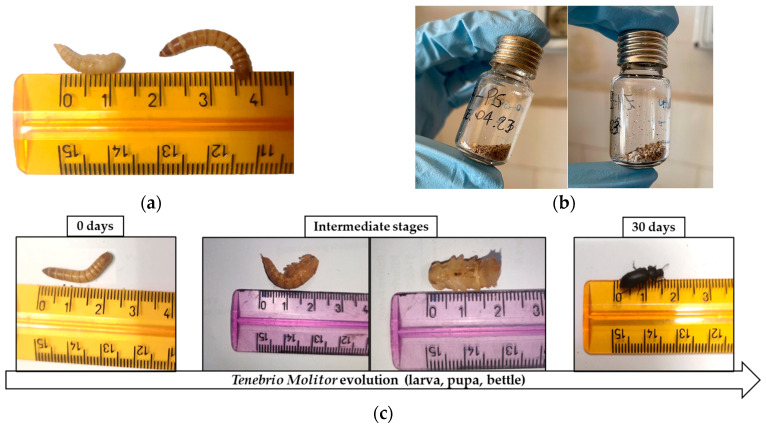
(**a**) Length measurement of the pupa and larva of *T. Molitor*; (**b**) frass of *T. Molitor* and *Z. Morio* larvae collected at the end of the experiment (30 days) after feeding on polystyrene, and (**c**) *T. Molitor* evolution from larvae to pupa/beetles for more than ca. 30 days.

The mealworms were fed with polystyrene blocks (PS) and polystyrene treated with H_2_O_2_ and microwave irradiation (PS-H_2_O_2_) as their sole diet for 30 days. Figure 1a shows the difference in length of the *Tenebrio Molitor* larva before and after its transformation into a pupa. As mealworm pupae are not mobile, removing the dead larvae and pupae from the containers during the experiment was performed to prevent them from being eaten and to protect them from cannibalism by the remaining larvae.

Frass samples were collected from the container after 15 and 30 days of the experiment and Figure 1b shows the difference between *Tenebrio Molitor* frass and *Zophobas Morio* frass collected after 30 days.

Figure 1c shows the evolution of *T. Molitor* from larva to pupa/beetle for more than ca. 30 days, using real images of *T. Molitor*’s evolution stages. It is important to highlight that after ca. 30 days, all larvae turned into pupae/beetles, and the experimental analysis, concerning the nutritional diet with bran or plastic, was inevitably interrupted.

#### 2.3.1. Weight Variation and Survival Rate

The weight of the larvae was monitored by weighing them every 5 days and ended on day 30. Since not all larvae were the same size, the weight was normalized to the number of individuals and the NW, normalized weight (grams of total larvae/number of larvae), was calculated. The tests were conducted in triplicate.

#### 2.3.2. Size-Exclusion Chromatography (SEC) Analysis

The changes in the molecular weight of the polymer were assessed by SEC. The detection was carried out using a differential refractometer. THF was the mobile phase with a flow rate of 1 mL min^−1^. Calibration was performed with polystyrene standards (from 20,250 to 470,000 g mol^−1^). Preparative SEC analyses were performed in THF with Azura GPC Knauer apparatus equipped with four TSKgel Guard Super columns using an RDI 2.1 L differential refractometer. In total, 60 microliters of a polymer solution (3 mg mL^−1^) were injected and eluted at a flow rate of 1 mL min^−1^.

#### 2.3.3. Spectroscopy Analysis

A Fourier Transform Infrared Spectrometer (Spectrum One, Perkin Elmer, Shelton, CT, USA) was used to record IR spectra using 16 scans at a resolution of 4 cm^−1^ in the range 4000–450 cm^−1^, using air as the background. For KBr pelleting sample preparation, a press was used to produce KBr pellets of the powder. The frass samples were mixed with KBr at a ratio of 1:100 and ground in a mortar and pestle to prepare a homogeneous powder. The mixture was then pressed into a 7 mm pellet using a hydraulic press (Mini Pellet Press, Specac, Orpington, UK). 

#### 2.3.4. Thermogravimetric Analysis (TGA)

The thermal stability of the entire sample series was confirmed using a TGA 8000 apparatus (Perkin Elmer Milan—Italy). The analyses were performed on the samples with a nitrogen flow of approximately 60 mL/min, with a heating ramp of 10 °C/min, in the temperature range from 50 °C to 600 °C. Data were acquired and analyzed using Pyris Software V. 13.3.3.0032, provided by the manufacturer.

#### 2.3.5. Morphological Analysis

The morphology of the surfaces of PS, PS-H2O2 and frass samples were investigated using a Scanning Electron Microscope (SEM FEI, Ocala, FL, USA, Quanta 200 FEG) equipped with an X-ray energy-dispersive spectrometer (EDS). Prior to examination, samples were placed onto a conductive stub and then gold-sputtered to avoid electrostatic charging effects.

## 3. Results and Discussion

### 3.1. Weight Variation and Survival Rate of T. Molitor and Z. Morio larvae

**Figure 2:**
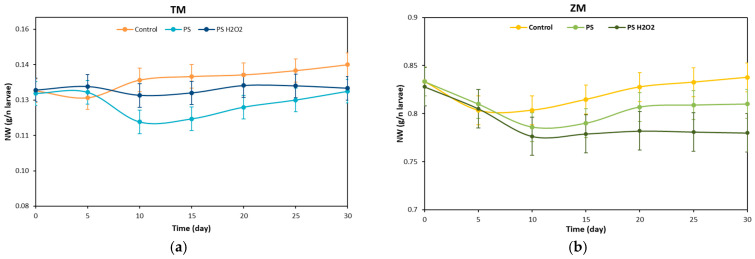
Weight variation during the time: (**a**) NW of *T. Molitor* and (**b**) NW of *Z. Morio* fed with bran (control), foam PS and foam PS-H_2_O_2_.

The weight of the larvae was monitored every 5 days during the test and Figure 2a,b show the normalized weight (NW) of the *T. Molitor* and *Z. Morio* larvae over 30 days of testing.

The NW of the *T. Molitor* larvae eating bran slightly decreased during the first 5 days; see Figure 2a. The NW then slowly increased over the further 25 days, reaching a final increment of ~8.5% with respect to the initial NW value. The NW of *T. Molitor* larvae eating PS remained almost constant during the first 5 days, while it decreased at 10 days (time requested to assess and adapt to the conditions). The NW then increased during the remaining 20 days with a final NW value similar to the initial value. *T. Molitor* larvae eating PS treated with H_2_O_2_ maintained a similar NW value to the initial NW value all over the 30 days, suggesting that larvae ate the PS-H_2_O_2_ foams more easily than the untreated ones. These results are in line with the data available in the literature. Peng et al. [23] reported a mass decrease of ~8% for *T. Molitor* larvae eating PS, while the same authors observed a mass increase of ~14% when feeding the larvae with PS and bran at the same time. 

The NW of the *Z. Morio* larvae eating bran showed a similar trend to that of the *T. Molitor* ones; see Figure 2b. However, a lower final NW value of ~0.5% was achieved at 30 days. Conversely, the NW of the *Z. Morio* larvae eating treated and untreated PS foams always decreased during the first 10 days, by ~6%. The NW of larvae eating PS then slightly increased, while that of larvae eating PS-H_2_O_2_ remained almost constant. The final NW values of larvae fed with untreated and treated PS were ~2.8% and ~5.8% lower than the initial value (day 0). 

**Figure 3:**
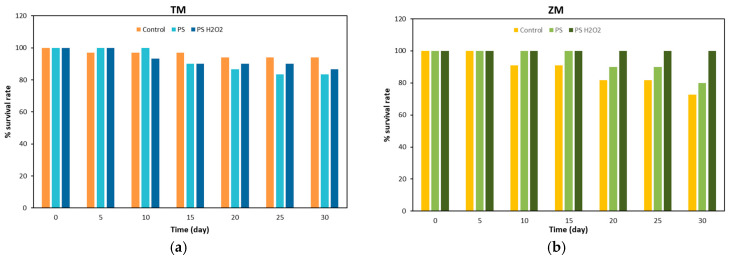
Survival rate of (**a**) *T. Molitor* and (**b**) *Z. Morio* larvae fed with bran (control), foam PS and foam PS-H_2_O_2_.

The survival rates of the larvae of *T. Molitor* and *Z. Morio* eating bran (control), PS foam and PS-H_2_O_2_ foam are shown in Figure 3 and summarized in Table 1. 

In the case of *T. Molitor* larvae, see Figure 3a, the larvae feeding on bran showed the highest survival rate of ~94% at 30 days. Conversely, the larvae fed PS reported the lowest value of ~83%. The larvae fed with PS-H_2_O_2_ foam had a survival rate of ~87%. In the literature, Peng et al. [23] reported survival rates of ~90% for *T. Molitor* larvae eating PS, while Yang et al. [19] observed values of ~86%. In addition, Yang et al. [19] also measured survival rates of ~85% for larvae eating bran. The different, although similar, survival rates of *T. Molitor* larvae can be attributed to the genetic differences between mealworm populations around the globe [24].

In the case of the *Z. Morio* larvae, the highest survival rate was exhibited by larvae eating PS-H_2_O_2_. The lowest value of ~73% was found for *Z. Morio* larvae eating bran, while the larvae fed with PS had a survival rate of ~80%. These results are in contrast with the NW behavior of *Z. Morio* larvae, indicating that, although the larvae lost weight during the tests, they had a more suitable environment where they lived. The lower survival rates of the *Z. Morio* larvae with respect to that of the *T. Molitor* ones were somehow expected. An et al. [3] reported a survival rate of ~70% for PS-foam-eating and normal diet (bran)-eating *Zophobas atratus* larvae, which belong to the family of *Zophobas larvae*.

### 3.2. Size-Exclusion Chromatography (SEC) Analysis

**Figure 4:**
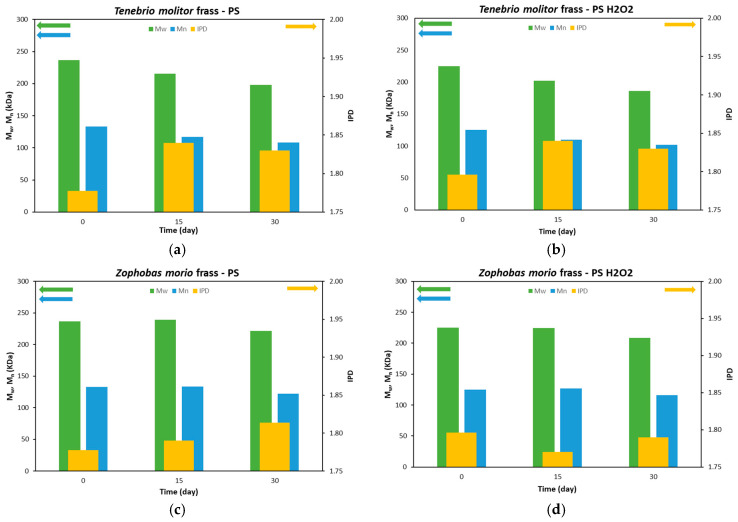
Data related to the changes in ponderal molecular weight (M_w_) (green, left axis), numerical molecular weight (M_n_) (blue, left axis) and polydispersity index (IPD) (yellow, right axis) of PS and PS-H_2_O_2_ (0 days), and frass of (**a**,**b**) TM and (**c**,**d**) ZM after 15 and 30 days.

SEC analysis was conducted to characterize the depolymerization and biodegradation of ingested PS [25]. The ponderal molecular weight (M_w_), numerical molecular weight (M_n_) and polydispersity index (IPD) of frass of *T. Molitor* and *Z. Morio* after 15 and 30 days are shown in Figure 4a,b and Figure 4c,d, respectively. The M_w_, M_n_ and IPD of PS and PS-H_2_O_2_ foams analyzed at day 0 are also reported. 

The M_w_ and M_n_ of PS frass of *T. Molitor* larvae decreased over 30 days. Specifically, M_w_ was 237, 215 and 197.8 kDa at 0, 15 and 30 days, respectively, with a final reduction of 16.4%. M_n_ values decreased from 133, 117 to 108.4 kDa, again showing a decrease of 18.6%. In the literature, Yang et al. [19] reported similar M_w_ and M_n_ percentage reductions. Peng et al. [23] also observed an M_n_ decrease of ~20%; however, the authors reported an M_w_ reduction of ~60%. IPD increased in digested samples, as a consequence of the depolymerization and degradation action of the larvae producing lower-molecular-weight fragments [19,26]. PS-H_2_O_2_ foams already had lower initial values of M_w_ and M_n_ with respect to those of pristine PS ones due to the action of the H_2_O_2_, which also caused an increase in the IPD index. M_w_ and M_n_ decreased in the frass of *T. Molitor* larvae eating PS-H_2_O_2_ foam due to the action of the larvae’s digestion, showing similar reduction percentages to those of the PS foam case; see Figure 4b. 

A lower degradation action was observed in *Z. Morio* larvae. The M_w_ and M_n_ values remained almost constant during the first 15 days in the frass from both PS and PS-H_2_O_2_ foams, while a decrease of ~7% was only achieved at 30 days; see Figure 4c,d. The results indicate the superior depolymerization and biodegradation activity of *T. Molitor* larvae compared to *Z. Morio* larvae.

### 3.3. Spectroscopy Analysis

**Figure 5:**
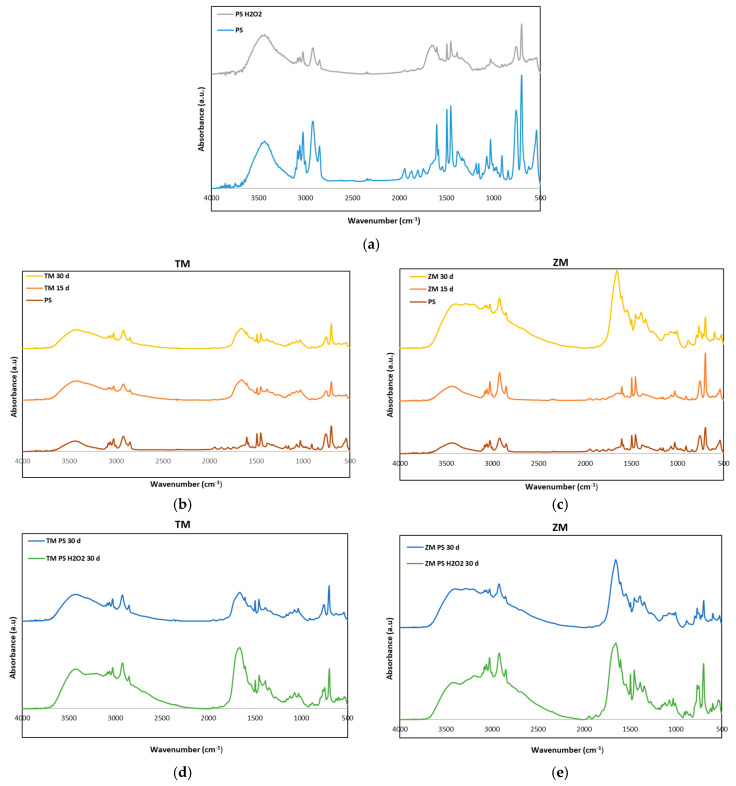
FTIR spectra of (**a**) neat PS and PS-H_2_O_2_ before the experiments (0 days) and FTIR spectra of (**b**) frass of TM and (**c**) frass of ZM for the larvae fed with PS after 15 and 30 days. (**d**,**e**) are the comparison of the spectra after 30 days for frass of TM and ZM feeding on PS and PS-H_2_O_2_, respectively.

The FTIR spectra of neat PS and PS-H_2_O_2_ foams before and after *T. Molitor* and *Z. Morio* larvae degradation are shown in Figure 5. Specifically, Figure 5a illustrates the FTIR spectra of neat PS and PS-H_2_O_2_ foams; Figure 5b,c refer to the frass collected after 15 and 30 days from *T. Molitor* and *Z. Morio* larvae eating PS foams; and Figure 5d,e report the comparison between FTIR spectra of frasses collected at 30 days from *T. Molitor* and *Z. Morio* larvae eating PS and PS-H_2_O_2_ foams.

Peaks at 625–970 cm^−1^ (ring-bending vibration) were visible in all FTIR spectra, although the peak intensity was slightly damped after larvae digestion. The characteristic peaks known to represent the PS benzene ring (1550–1610 cm^−1^ and 1800–2000 cm^−1^) were visible in the neat PS samples, while they were considerably dampened in frass, providing evidence of ring cleavage. In addition, evidence of degradation was the appearance of carbonyl groups (1700 cm^−1^) in frass. The broadening of peaks at 2500–3500 cm^−1^ in the FTIR of frass was also associated with the hydrogen bond of hydroxyl groups and/or carboxylic acid groups, suggesting a shift from hydrophobic to more hydrophilic surface properties [12]. 

The frass of *T. Molitor* larvae showed PS degradation already after 15 days; Figure 5b. Conversely, the frass of *Z. Morio* larvae after 15 days had the same FTIR spectrum as neat PS foams. The H_2_O_2_ treatment already caused a damping effect of peaks at 2500–3500 cm^−1^, as well as of those at 1550–1610 cm^−1^ and 1800–2000 cm^−1^, indicating the degradation of the PS material [20,22]. Treatment of the PS with H_2_O_2_ promoted the degradation action of the larvae. A more intense peak at 1700 cm^−1^ and a wider peak at 2500–3500 cm^−1^ were, in fact, reported in FTIR spectra of frass collected from larvae eating PS-H_2_O_2_ foams with respect to those collected from the PS-eating ones; see Figure 5d. 

### 3.4. Thermogravimetric Analysis

**Figure 6:**
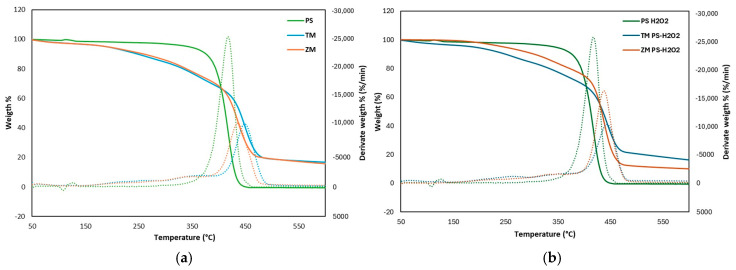
TGA curve of (**a**) PS and (**b**) PS-H_2_O_2_ and frass of *T. Molitor* and *Z. Morio* larvae after 30 days feeding on foam PS and PS-H_2_O_2_. Solid lines represent weight curve (left axis) and dashed lines represent DTG curve (right axis).

Thermogravimetric analysis (TGA) can be considered a valuable tool to compare the changes in the composition and the modification by larvae from pristine foam PS to frass at the end of the test (30 days). As noticeable in Figure 6a,b, the TGA curves of PS and PS-H_2_O_2_ showed only one sharp mass loss where more than 95% of the loss occurred between 350 °C and 460 °C, and specifically, the maximum decomposition rate occurred at ca. 420 °C for both PS and PS-H_2_O_2_. 

In contrast, the frass of *T. Molitor* fed only PS showed three weight loss stages: stage 1 of 10% at 150–300 °C (max. decomposition rate occurred at 260 °C), stage 2 of 17.5% at 300–395 °C (max. rate at 364 °C) and stage 3 of 43.0% at 395–500 °C (max. rate at 451 °C). When *Z. Morio* was fed only PS, the frass showed three weight loss stages: stage 1 of 6.1% at 150–260 °C (max. rate at 207 °C), stage 2 of 14.0% at 260–373 °C (max. rate 338 °C) and stage 3 of 44.0% at 373–487 °C (max. rate 434 °C). Similarly, the frass of both *T. Molitor* and *Z. Morio* fed PS-H_2_O_2_ showed three weight loss stages with maximum decomposition rates very similar to those fed with PS in Figure 6a, although there is a small difference between TM-PS-H_2_O_2_ and ZM-PS-H_2_O_2_. The frass of TM fed with PS-PS-H_2_O_2_ showed the lowest derivative weight, indicating that the PS content in the frass was lower and the degradation efficiency of PS was higher.

According to the literature [5,13,19,23], under the same heating program, the frass of TM or ZM decomposed in more stages than the control. This suggests that the frass contained not only PS but also other components produced during digestion in the insect gut. During stage 3, the weight loss of the frass was less than the control, demonstrating the depletion of PS content in the frass. 

### 3.5. Morphological Analysis

**Figure 7:**
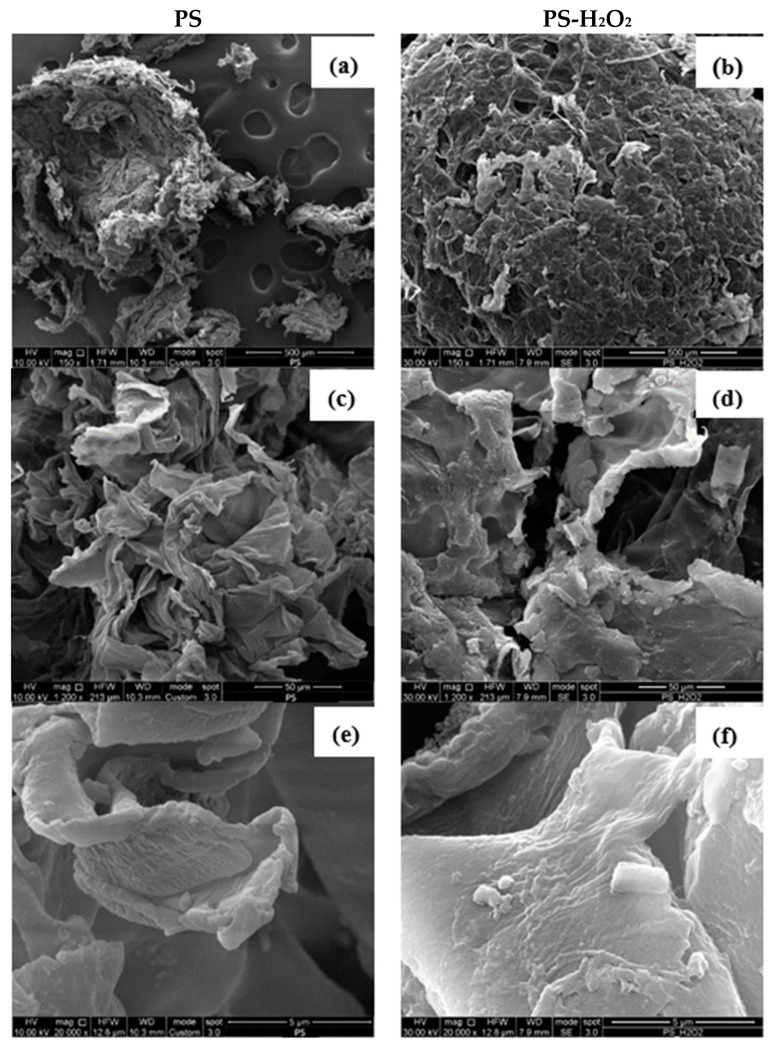
SEM observations at different magnifications, i.e., 150×, 1200× and 20,000×, of PS (**a**,**c**,**e**) and PS-H_2_O_2_ (**b**,**d**,**f**) frass samples, respectively.

The surface erosion of the plastic by the larvae was evaluated by SEM analysis of the frass. Firstly, the action of H_2_O_2_ and microwave irradiation on the PS foam was investigated. Figure 7 shows the SEM images of the original PS samples and those treated with PS-H_2_O_2_ at different magnifications, i.e., 150×, 1200× and 20,000×.

A small sphere of PS foam can be seen in Figure 7a, which probably imploded due to the high vacuum pressure applied during the gold sputter process. At a higher magnification, in Figure 7c,e, the smooth surfaces of the PS material can be clearly seen, as typically reported in the literature [24]. Similar structures and smooth surfaces can also be observed in the PS-H_2_O_2_ samples; Figure 7b,d,f. This indicates that the action of H_2_O_2_ did not affect the surface of the PS foam. Based on this result, only the SEM images of the frond collected after 15 and 30 days from *T. Molitor* and *Z. Morio* larvae that fed on PS foam will be discussed below.

**Figure 8:**
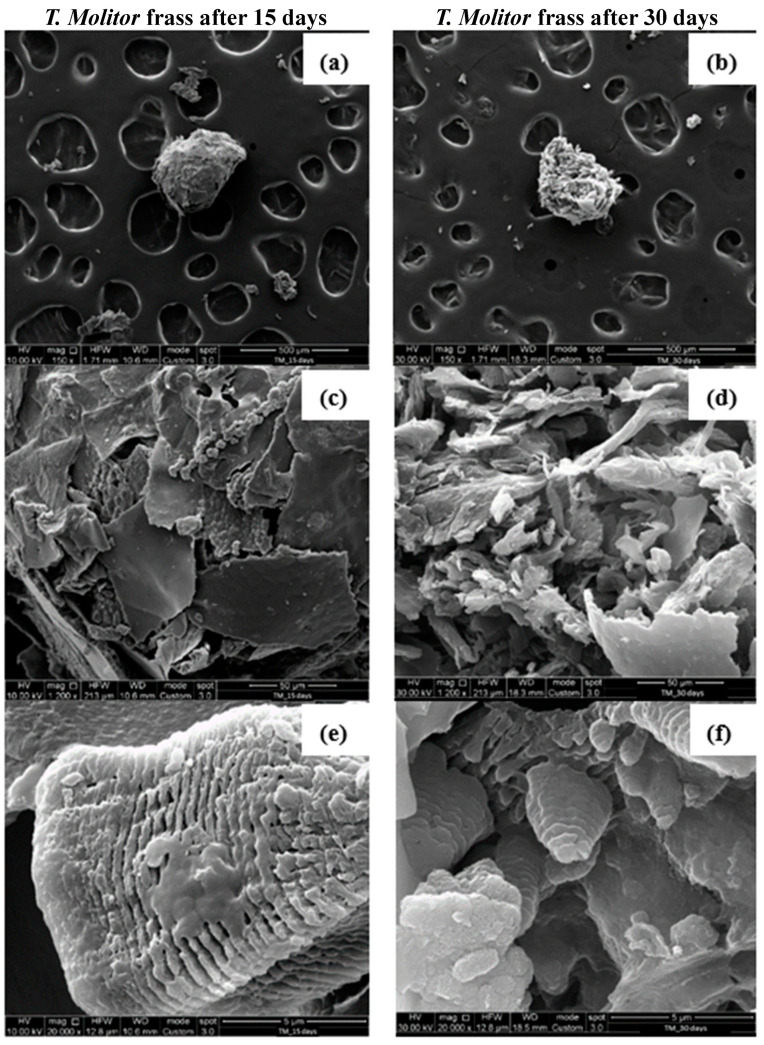
SEM observations at different magnifications of *T. Molitor* frass after 15 (**a**,**c**,**e**) and 30 days (**b**,**d**,**f**) of PS foam feeding.

Figure 8a,c,e are the SEM images of *T. Molitor* frass after 15 days, while Figure 8b,d,f refer to the SEM images of *T. Molitor* frass after 30 days. PS samples isolated from the frass of *T. Molitor* larvae showed completely different, rough and irregularly carved surface structures from the PS foam; see Figure 7.

PS flakes can be observed at a magnification of 1200×, Figure 8c,d. In addition, pores can be identified at a magnification of 20,000× on the PS surface, Figure 8e,f, demonstrating the degradation action of the larvae. Surface alterations in the plastics are characteristic of the aging processes occurring under the influence of microorganisms present in the intestines of insects or, more specifically, the enzymes secreted by them. The folding of the previously smooth surface of the polymer and the formation of pitting were observed during the biodegradation of the polymers [24].

**Figure 9:**
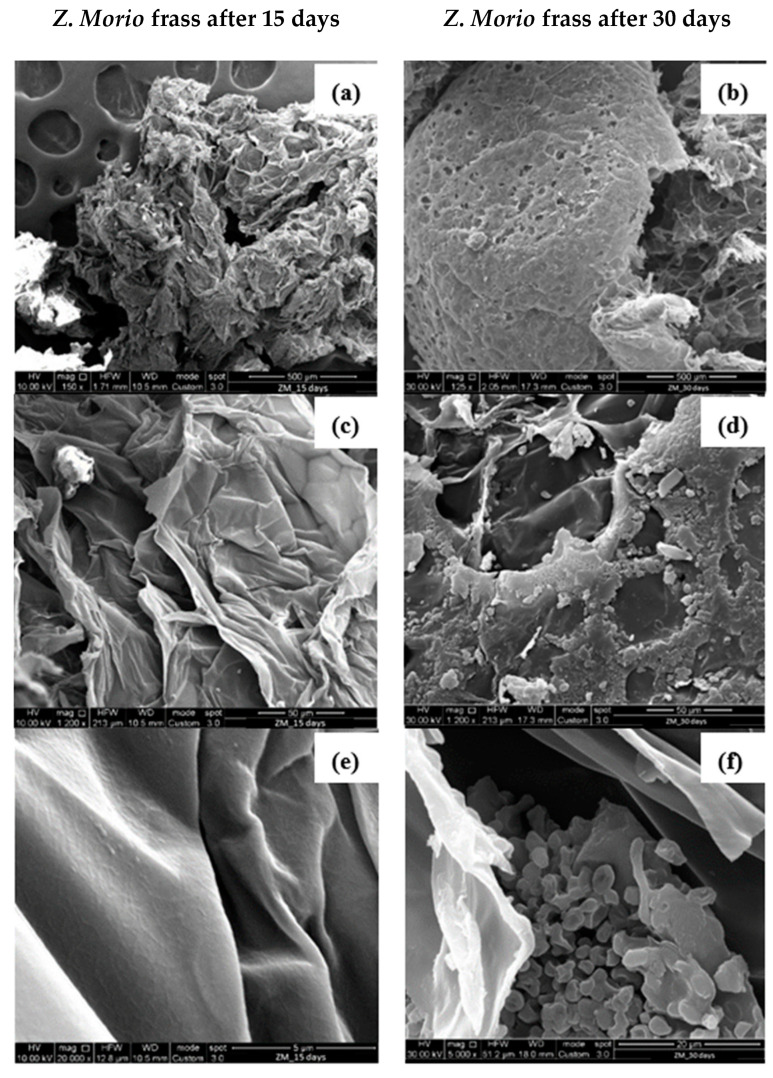
SEM observations at different magnifications of *Z. Morio* frass after 15 (**a**,**c**,**e**) and 30 days (**b**,**d**,**f**) of PS foam feeding.

SEM observations of frasses collected from *Z. Morio* larvae after 15 and 30 days are shown in Figure 9. 

The SEM images of PS samples collected from the frass of the *Z. Morio* larvae after 15 days, Figure 9a,c,e, showed a very similar structure with respect to that of the PS one, Figure 7. This indicates that *Z. Morio* larvae did not interact with the PS material during the first 15 days of the tests, as also discussed in Section 3.1, Section 3.2, Section 3.3 and Section 3.4.

**Figure 10:**
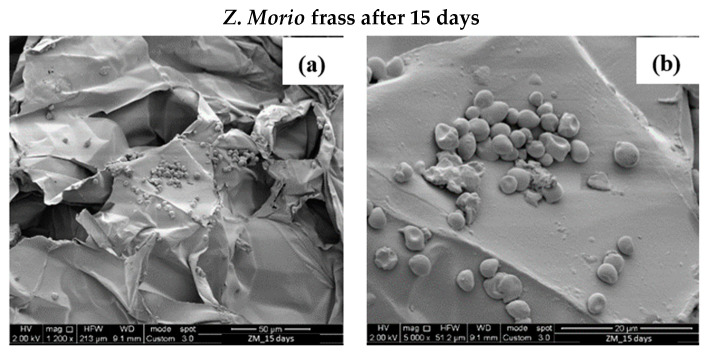
(**a**,**b**) SEM observation at different magnifications of organic matter in the frass of *Z. Morio* larvae after 15 days fed with PS foam.

PS degradation can be noticed only after 30 days, namely, PS shreds and holes can be observed Figure 9b,d,f. However, comparing Figure 8b and Figure 9b, it can always be observed that large pieces of PS materials are still found in the frass of *Z. Morio* larvae, confirming the lower degradation action of the *Z. Morio* larvae compared to *T. Molitor* larvae. Interestingly, traces of microorganisms were observed in the frass of Z. *Morio* larvae as shown in Figure 10.

Spherical cocci bacteria are observed. The presence of cocci bacteria is in accordance with evidence reported by Yang et al. [27]. The authors attributed the ability of the *Z. Morio* larvae to depolymerize PS to the presence of microbiota, such as the cocci, in their gut. Cocci bacteria were also found in frass after 30 days of tests, here omitted for the sake of brevity.

## 4. Conclusions

In this work, the biodegradation of PS by *T. Molitor* and *Z. Morio* has been investigated, also considering the treatment of PS with H_2_O_2_ in order to facilitate PS degradation. As discussed before, six different experimental diets were compared: (i) *T. Molitor* fed with bran; (ii) *T. Molitor* fed only PS; (iii) *T. Molitor* fed only PS treated with H_2_O_2_; (iv) *Z. Morio* fed with bran; (v) *Z. Morio* fed only PS; and (vi) *Z. Morio* fed only PS treated with H_2_O_2_. The change in the larvae mass and the survival rate were monitored periodically and the obtained results suggest that both *T. Molitor* and *Z. Morio* larvae are able to biodegrade the PS and their mass changes and survival rate are similar to those of larvae fed with bran. The analysis of *T. Molitor* and *Z. Morio* frass after 15 and 30 days shows a slightly decreased PS molecular weight and an increase in polydispersity, suggesting a reduction in PS chain weight and length. Further, the data coming from FTIR analysis support this thesis, highlighting the changes in PS compositions after digestion. 

Therefore, all these results highlight the ability of both larvae to survive using plastic as feed rather than bran.

Therefore, the identification and knowledge of the enzymes involved could open a new window for the controlled degradation and depolymerization of polymers formulated by polyaddition.

## Figures and Tables

**Table 1:** Summary of PS biodegradation by *T. Molitor* and *Z. Morio* larvae.

Worm Source	Initial Weight (g)	Feedstocks	Survival Rate (%)	M_w_ of Frass (kDa)	M_w_ Reduction (%)	M_n_ of Frass (kDa)	M_n_ Reduction (%)	PDI
*T. Molitor*	3.8	Bran	94	n.d.	n.d.	n.d.	n.d.	n.d.
		PS	83	197.8	16.4	108.4	18.6	1.83
		PS-H_2_O_2_	87	186.0	17.3	101.8	18.7	1.83
*Z. Morio*	8.3	Bran	73	n.d.	n.d.	n.d.	n.d.	n.d.
		PS	80	221.9	6.3	122.3	8.1	1.81
		PS-H_2_O_2_	100	208.5	7.3	116.2	7.1	1.79

Note: n.d. means not determined.

## Data Availability

Data are contained within the article.
